# GRMA-Net: A novel two-stage 3D Semi-supervised Pneumonia Segmentation based on Dual Multiscale Uncertainty Estimation with Graph Reasoning in Chest CTs

**DOI:** 10.2174/0115734056363870250804215311

**Published:** 2025-08-08

**Authors:** Jianning Zang, Yu Gu, Lidong Yang, Baohua Zhang, Jing Wang, Xiaoqi Lu, Jianjun Li, Xin Liu, Ying Zhao, Dahua Yu, Siyuan Tang, Qun He

**Affiliations:** 1 Inner Mongolia Key Laboratory of Pattern Recognition and Intelligent Image Processing, School of Digital and Intelligent Industry, Inner Mongolia University of Science and Technology, Baotou, 014010, China; 2 School of Automation and Electrical Engineering, Inner Mongolia University of Science and Technology, Baotou, 014010, China; 3 School of Information and Electronics, Beijing Institute of Technology, Beijing 100081, China; 4 School of Computer Science and Technology, Baotou Medical College, Inner Mongolia University of Science and Technology, Baotou, 014040, China; 5 School of Humanities and Law, Inner Mongolia University of Science and Technology, Baotou, 014010, China

**Keywords:** Semi-supervised Learning, Uncertainty estimate, Graph reasoning, Medical image segmentation, Mean teacher, NSD

## Abstract

**Introduction:**

This study aims to propose and evaluate a two-stage semi-supervised segmentation framework with dual multiscale uncertainty estimation and graph reasoning, addressing the challenges of obtaining high-precision pixel-level labels and effectively utilizing unlabeled data for accurate pneumonia lesion segmentation.

**Methods:**

First, we design a guided supervised training strategy for modeling aleatoric uncertainty (AU) at dual scales, reducing the impact on segmentation performance caused by aleatoric uncertainties introduced by blurred lesions and their boundaries in the image. Second, we design a training strategy for multi-scale noisy pseudo-label correction to reduce the cognitive bias problem caused by unreliable predictions in the model. Finally, we design a new combination of fused feature interaction graph reasoning (FIGR) and attention modules, which enables the network model to better capture image features in small infected regions.

**Results:**

Our study was validated using the MosMedData public dataset. The proposed algorithm improves the performance by 1.25%, 1.03%, 2.98%, and 0.59% on Dice, Jaccard, normalized surface dice (NSD), and average distance of boundaries (ADB), respectively, compared to the baseline model.

**Discussion:**

Our semi-supervised pneumonia segmentation framework, through two-stage multi-scale uncertainty estimation and modeling, significantly improves segmentation performance by leveraging unlabeled data and addressing uncertainties, offering clinical benefits in pneumonia diagnosis while facing challenges in generalization and computational efficiency that future work will target with GAN-based data synthesis and architecture optimization.

**Conclusion:**

It can be convincingly concluded that the proposed algorithm is of profound importance and value in the domain of clinical practice.

## 1 INTRODUCTION

Pneumonia is an acute respiratory infection caused by viruses and bacteria, commonly found in children under 5 years old and elderly people with chronic diseases [1, 2]. Pneumonia affects 7% of the world's population [3], which means that approximately tens of thousands of patients are at serious health risk. Therefore, a timely and accurate diagnosis of pneumonia is indispensable to provide effective treatment to patients [4]. Physicians usually determine the presence of an infection in the lungs based on imaging features demonstrated on computed tomography (CT) scans, it is difficult for physicians to determine the presence of an infection in the lungs due to the small size of the infected area in a patient's lungs in the early stages of life and the low contrast with normal tissue, which can lead to misdiagnosis. Patient's chest CT images diagnosed with pneumonia are shown in Fig. (**1**). In recent years, computer-aided diagnosis (CAD) systems have been increasingly used in the fields of medical image analysis and radiology [5-10]. CAD systems can be combined with CT images to assist physicians in clinical decision making and improve diagnostic efficiency and accuracy [11]. Among them, image segmentation is an important part of CAD. Image segmentation separates and marks the infected region to enable further analysis and discrimination of the CAD system.

Supervised deep learning based methods perform well in medical image segmentation; however, the excellent results of these methods are achieved by training with a large amount of labeled data [13]. In contrast to natural images, infected areas in medical images need to be manually labeled by specialized radiologists. CT images can reveal irregular variations in the size, location, and morphology of infected areas in the lungs, as well as small differences from normal tissue, resulting in blurred lesion areas and their boundaries [14]. Labeling this data takes a lot of time for the radiologist. Therefore, it is very difficult to obtain a large amount of high-precision labeled medical data for deep learning model training. Recently, the task of medical image segmentation based on semi-supervised learning has received increasing attention [15]. Different from supervised learning, semi-supervised learning can mine useful information from unlabeled image data and combine it with limited labeled data for training to improve the robustness and generalization ability of the model, which plays an important role when data is scarce or difficult to label. Related scholars have conducted outstanding research on semi-supervised segmentation. Tarvainen *et al*. [16] addressed the shortcomings of temporal ensembling by proposing a mean teacher model based on consistent regularization, whose framework is shown in Fig. (**2a**). Yu *et al*. [17] proposed an uncertainty-aware (UA) self-ensembling model. The teacher model uses Monte Carlo methods to estimate uncertainty regions for guiding consistency loss, allowing students to learn from low-uncertainty objectives. The UA-MT framework is shown in Fig. (**2b**). Lu *et al*. [18] guided the consistency loss with noisy pseudo-labels by estimating uncertainty from the predictions of teacher-student models. Xiang *et al*. [19] fused cognitive uncertainty and aleatoric uncertainty for the first time to guide training. Lei *et al*. [20] introduced an adversarial consistency approach to the mean teacher model, which enhances the ability of the segmentation network to transfer knowledge between labeled and unlabeled data. Wu *et al*. [21] introduced a cross-patch dense contrastive learning method based on the mean teacher model, enhancing the inter-class separability and intra-class compactness of features. Wang *et al*. [22] enabled student models to learn from reliable targets by introducing triple uncertainty and an uncertainty-weighted integration strategy accounting for variability between sampling outcomes. In contrast to previous methods, we propose a semi-supervised pneumonia segmentation method with dual multiscale uncertainty estimation and modeling, and graph reasoning. This method is illustrated in Fig. (**2c**).

As semi-supervised training strategies are increasingly applied to medical image segmentation tasks, the problem of insufficient labeled data is alleviated by utilizing unlabeled data, but the following problems still exist. First, although there are studies that have improved the reliability of model-generated pseudo-labels through a number of strategies, however, they do not consider the problem that models may generate noisy or unreliable pseudo-labels at multiple scales. Second, in semi-supervised training, the limited labeled image data can have a significant impact on the overall segmentation results of the model, while most of the research has ignored the problem of aleatoric uncertainty or stochasticity caused by fuzzy infected regions in supervised training. Finally, most of the networks in semi-supervised medical image segmentation-based tasks are constrained to small convolutional kernels, leading to the problem of limited receptive field. To address these limitations, we propose a semi-supervised pneumonia segmentation framework based on a two-stage dual multiscale uncertainty estimation and graph reasoning based on the mean teacher model.

In the supervised training phase, a new guided supervised training strategy for modeling aleatoric uncertainty (AU) at two scales is designed, efficiently capture inter-pixel correlations at both scales by modeling aleatoric uncertainties as multivariate normal distributions in logit space. The model is enabled to generate multiple possible predictions, which helps reduce the influence of blurred lesions and their boundaries in the image on segmentation performance.

In the unsupervised training phase, a training strategy for multi-scale pseudo label correction learning was designed. In order to reduce the impact of generating noisy pseudo-labels for unlabeled data on segmentation performance and the problem of cognitive bias on the model. The multi-scale output predictions at the decoder end of the teacher-student models are computed separately as Kullback-Leibler (KL) variances and used as uncertainty estimates to guide the loss of consistency of pseudo-labeling inclusion at the corresponding scales.

In addition, we design a new combination of fusing feature interaction graph reasoning (FIGR) and attention module, graph reasoning for features along channels in teacher-student models. The features of refined relational perception are obtained through three phases: graph projection, graph reasoning, and graph reprojection. This method effectively models dependencies between distant pixels and fuses contextual information.

## 2 METHODS

### 2.1 Semi-supervised Segmentation Framework Design

Based on the DM^2^T-Net [23], this study proposes a two-stage 3D semi-supervised pneumonia segmentation method based on dual multiscale uncertainty estimation with graph reasoning. The structure of this segmentation framework is shown in Fig. (**3**). Multi-scale based uncertainty estimation was designed to guide the training in the supervised and unsupervised phases, respectively. In the proposed semi-supervised framework, a Mean Teacher model is used, which consists of a teacher model and a student model of the same network structure. We use the proposed the Graph Reasoning and Multi-dimensional Attention Network (GRMA-Net) as teacher network and student network, the structure of GRMA-Net is shown in Fig. (**4**). The GRMA-Net in the framework is based on the improvement of the multiple-dimensional attention convolutional neural network (MDA-CNN) [23]. In addition, the structure of the attention module in the network is shown in Fig. (**5**).

During the supervised training phase, labeled data is fed to the student model, which generates predictions for four different scales on the decoder side. Subsequently, the model computes the supervised loss between the different scales to impose consistency constraints on the multiscale outputs. In addition to this, the aleatoric uncertainty (AU) module of the supervised training phase utilizes a low-rank covariance matrix on both scales by constructing a multivariate normal distribution in logit space in order to efficiently model the aleatoric uncertainty introduced by ambiguous lesions and their boundaries in the image. In the unsupervised training phase, unlabeled data is input into the teacher-student models. The predictions of teacher-student models at different scales are used to calculate KL-variance and serve as uncertainty estimation to guide consistency loss with noisy pseudo-labels. The details of the rationale for aleatoric uncertainty modeling to guide training, uncertainty estimation guides consistency loss with noisy pseudo-labels, and the FIGR module are described in detail in 2.3, 2.4, and 2.5, respectively.

### 2.2 GRMA-Net Structure Design

The GRMA-Net shown in Fig. (**4**) serves as the backbone segmentation network for the proposed two-stage 3D semi-supervised pneumonia segmentation approach. Based on 3D CNN, the network can fully leverage contextual information in 3D space to generate more accurate and discriminative features than 2D CNN networks [24]. On the encoder side, images Q and G are initially obtained by down-sampling the input 3D image F in two different spatial dimensions. Subsequently, the 3D images F, Q, and G at different spatial dimensions are processed through a series of convolution and maximum pooling operations to generate the feature maps *F_i_*, *Q_i_*, and *G_i_*. Following each set of features, the first layer of the network aggregates feature maps of different spatial resolutions through an attention module, while feature maps of the same spatial resolution in other layers are aggregated through an attention module. The structure of the attention module is shown in Fig. (**5**). In addition, in the final down-sampling layer, the FIGR module is integrated before the feature map input attention module, which performs graph reasoning on the features along the channel dimensions and can capture global contextual dependencies. Finally, in the decoder stage, up-sampling of features is performed before merging with adjacent feature maps, then going through the 3×3×3 CNN layers, 1×1×1 CNN layer, respectively. The final output predictions at different scales are obtained.

Fig. (**5**) shows a schematic diagram of the attention module. For the three feature maps *F_i_*, *Q_i_*, and *G_i_*, the feature map *Q_i_* undergoes global average pooling (GAP), a fully connected (FC) layer, and sigmoid operations, respectively, to obtain the feature map *A*_2_. Subsequently, the feature map *A*_2_ is multiplied by *F_i_* and then summed with *F_i_* to obtain the feature *H_i_*. For feature *G_i_* after GAP, FC layer, and sigmoid operations, the feature *A*_3_ is obtained, and the feature *A*_3_ is multiplied by *H_i_* and then added with *H_i_* to finally obtain the aggregated feature *AMF_i_*.

### 2.3 Aleatoric Uncertainty (AU) Modeling to Guide Training

The ambiguity and randomness present in medical images lead to the presence of aleatoric uncertainty [19]. Most current segmentation networks are fine-grained pixel classification tasks that classify each pixel in an image independently, ignoring inter-pixel correlation and contextual information, resulting in independent pixel-by-pixel estimates. In cases where lung lesions or their boundaries in image features are often ambiguous, the model should be able to consider multiple possible output scenarios. Xiang *et al*. [19] and Monteiro *et al*. [25] addressed this problem by modeling aleatoric uncertainty using multivariate normal distributions in the logit space, capturing inter-pixel dependencies through the estimated distributions while sampling the distributions multiple times to obtain the most likely output. Inspired by this, this study designs a semi-supervised learning framework that can model aleatoric uncertainty at two scales to guide supervised training, and the process of modeling aleatoric uncertainty is shown in Fig. (**6**).

The more reliable approach to the aleatoric uncertainty brought by ambiguous infection areas and their boundaries is to enable the model to capture this uncertainty and quantify it into a probability distribution form. Parameters computed by neural networks can be constructed as multivariate normal distributions to capture uncertainty, for the network input denoted by *x*, and before the softmax function is applied, it is assumed that the logit value satisfies a multivariate normal distribution, and the logit is denoted by *c*. The relationship between *x* and *η* is shown in Eq. (**1**) [19, 25].

**Table:** 

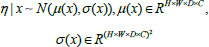	(1)

The equation shows that for a given input *x*, the variable *η* follows a multivariate normal distribution. Where *µ*(*x*) represents the mean value of *η*, with a size of *H×W×D×C, σ*(*x*), is the standard deviation of *η*, with a size of (*H×W×D×C*)^2^.

The two important parameters of the multivariate normal distribution are the mean and covariance matrix, which determine the shape and nature of the distribution, and these two parameters of this distribution can be calculated by a neural network. In addition, a low-rank, parameterized form of the covariance matrix is employed to approximate the covariance matrix in the high-dimensional space through a set of smaller parameters, which allows the neural network to efficiently compute the important parameters of the multivariate normal distribution as shown in Eq. (**2**).

**Table:** 

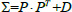	(2)

where ∑ denotes the covariance matrix in the low-rank parameterized form, P denotes the covariance factor, and D denotes the diagonal matrix.

This study models arbitrary uncertainty on two mutually independent scales of student model output prediction sizes 160×160×32 and 80×80×16, respectively. After constructing the multivariate normal distribution, due to the more complex form of the normal distribution, it may become difficult to compute the integral of the softmax function over the normal distribution. The Monte Carlo integration method is used to deal with the complex form of the integral, as shown in Eq. (**3**) [25].

**Table:** 

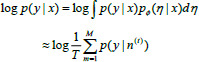	(3)

**Table:** 

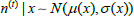	(4)

where *p*(*y* | *n*^(^*^t^*^)^) represents the logarithmic conditional probability of *y* given *n*^(^*^t^*^)^. *n*^(^*^t^*^)^ indicates that, given *x*, *n*^(^*^t^*^)^ follows a normal distribution with a mean of *µ*(*x*) and a standard deviation of *σ*(*x*). In Eq. (**4**), *µ*(*x*) and *σ*(*x*) are functions of *x*. M is the number of Monte Carlo samples. By performing a resampling operation on the computed distribution, through each sampling, a logit vector can be obtained, and the logit vector can be further transformed into a probability distribution [25]. After approximating a complex probability distribution by Monte Carlo integration, the probability distribution can be converted to a probabilistic output using softmax. Because of modeling arbitrary uncertainty on two independent scales, it is necessary to calculate the loss between the output probability value and the true value on these two scales separately and sum the loss on both scales. Based on the prior work described in [19], the loss function can be obtained as shown in Eq. (**5**).

**Table:** 

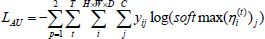	(5)

**Table:** 

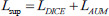	(6)

where p denotes the scale, this study sums the loss between the predicted and true values generated by the student model after modeling arbitrary uncertainty on two independent scales, 160×160×32 and 80×80×16, respectively. T denotes the number of iterations, H, W, and D denote the height, width, and depth of the data, and C denotes the category. *y_ij_* denotes the ground truth of the sample *i* on category *j*, *η*^(^*^t^*^)^*_i_* *η*^(^*^t^*^)^*_i_* denotes the predicted probability distribution in *t* iterations. *L_AUM_* is the cross-entropy loss between the computed predicted probability distribution and the ground truth at both scales. In Eq. (**6**), *L_DICE_* is the supervisory loss between the multiscale output of the student model and the ground truth. The final supervised loss in the supervised training phase is *L_DICE_+ L_AUM_*.

In addition, modeling arbitrary uncertainty as a multivariate normal distribution incurs high computational costs from covariance matrix operations. We adopt low-rank approximation (covariance factors and diagonal elements). As matrix dimension rises, distribution-creation and related computations grow complex. Additionally, Monte Carlo integration, which approximates integrals *via* resampling complex distributions, has computational complexity proportional to the number of samples, so overhead increases with more samples.

### 2.4 Uncertainty Estimation Guides Consistency Loss with Noisy Pseudo-labels

It is important to fully utilize unlabeled data in semi-supervised medical image segmentation tasks. The classical mean teacher model uses a consistency regularization method, which is considered a training strategy to improve the model's performance by generating pseudo-labels from sub-networks to each other after perturbing the inputs [26], at the same time, the model may also be affected by unreliable supervisory signals, leading to incorrect predictions. Lu *et al*. [18] simultaneously combine consistency regularization and pseudo-labeling to guide consistency loss with noisy pseudo-labels by estimating uncertainty. Inspired by this, we designed a training strategy to correct noisy pseudo-labels at multiple scales. We calculate the uncertainty of the output predictions from student-teacher models across different scales and use it as the KL-variance. This variance serves as an uncertainty estimation that guides the consistency loss with noisy pseudo-labels at various scales, ultimately reducing cognitive bias issues caused by unreliable predictions in the model.

In semi-supervised segmentation tasks, noisy or inaccurate pseudo-labels may be treated as correct targets, so the high quality of model-generated pseudo-labels is crucial for prediction results. On the other hand, for unlabeled data input to the model, the predictions should have a low entropy distribution [18], which is related to the implementation of pseudo-labeling through hard labeling methods [27]. Because the argmax operation on the output probability distribution generates a “one-hot” low entropy distribution [15]. Thus, draw on the previous study in [18], we apply pseudo-labeling in consistency loss and have the teacher model generate pseudo-labeling in multiple scale outputs. The argmax function is applied to each scale separately, effectively creating multiple “one-hot” representations. Based on the prior work described in [18], the loss of consistency between the pseudo-labeling of the teacher model output and the student model predictions at multiple scales can be represented by Eq. (**7**).

**Table:** 

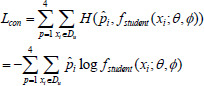	(7)

Where *x_i_* denotes the input image data, unlabeled data to be *x_i_* ϵ *D_u_*, ƒ*_student_* and ƒ*_teacher_* denote the student model and the teacher model, respectively. *θ* and *θ’* denote the parameters of the student and teacher models, respectively, and *φ* denotes Gaussian noise, *p* denotes the number of scales, *p^^^i* is the hard label obtained from the predicted output of the teacher model after the argmax function.

Combining pseudo-labeling and loss of consistency also has the unavoidable problem of uncertainty about the quality of pseudo-labeling. Lu *et al*. [18], Luo *et al*. [28], and Zheng *et al*. [29] explored the uncertainty in model output prediction. Inspired by this, the present study separately and simultaneously calculated the KL-variance between output predictions at different scales in the decoder side of the teacher-student models, and the KL-variance is used as uncertainty estimation to guide the consistency loss with pseudo-labels, thereby minimizing the impact of noisy pseudo-labels at each stage of the model. Relying on the previous study in [18], the computation of the KL-variance on multiple scales as an uncertainty estimation can be expressed in Eq. (**8**).

**Table:** 

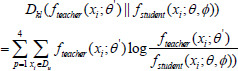	(8)

where ƒ*_student_*(*x_i_*; *θ*, *φ*) and ƒ*_student_*(*x_i_*; *θ*’) denote the output predictions of the student and teacher models, respectively. The meanings of ƒ*_student_* , ƒ*_student_*, *x_i_*, *θ*, *θ*’, *φ*, *p* and *D_u_* in the formula are the same as those in Eq. (**7**).

After the uncertainty estimation is obtained by calculating the KL-variance through the teacher-student models on multiple scales, we guide the consistency loss with noisy pseudo-labels at different scales. Therefore, based on the previous study in [18], the final consistency loss in the unsupervised stage of the semi-supervised segmentation framework can be represented by Eq. (**9**).

**Table:** 

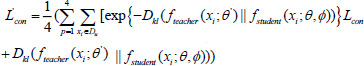	(9)

The meanings of ƒ*_student_*, ƒ*_student_*, *x_i_*, *θ*, *θ’*, *φ*, *p*, and *D_u_* in the formula are the same as those in Eq. (**7**). The formula indicates that the magnitude of KL-variance is related to the degree of penalization, and when the KL-variance is small, indicating that the predictions of the teacher-student models are very close, there is no need to penalize the consistency loss too much. When the KL-variance is large, it indicates that there is a discrepancy between the predictions of the teacher and student models, and the penalty for loss of consistency needs to be increased to induce the student model to better fit the predictions of the teacher model.

### 2.5 Feature Interaction Graph Reasoning Module

Most segmentation networks are constrained to a limited receptive field due to a small convolutional kernel, which makes it difficult for the network to capture long-range contextual dependencies between pixels and ultimately leads to inaccurate segmentation of the complex and variable lung infection region. Zhuang *et al*. [30] proposed dual-interaction volume modules that can perform graph reasoning in both spatial and channel dimensions to enhance network context dependency capture. Inspired by this, we designed a new combination of the fused feature interaction graph reasoning (FIGR) module and attention mechanism, which fuses FIGR in front of the attention module in the backbone segmentation network, which can make the network more conducive to capturing the dependencies between distant pixels. In addition, both the modeling of arbitrary uncertainty and the FIGR module boost segmentation models on complex data. modeling arbitrary uncertainty manages pixel-level uncertainty, offering stable pixel information for the feature interaction graph inference module. This enables the latter to better capture global context at the feature level.

The FIGR module is shown in Fig. (**7**). The FIGR module contains two key elements: node number and state number, where node number refers to the number of nodes in the graph and state number denotes the dimension of the features that each node has. The module consists of three parts: graph projection, graph reasoning, and graph reprojection. Through these three stages, the network can learn feature representations that are more favorable to the segmentation task.

Graph projection: To enable relational reasoning in graph space, the convolutional features need to be projected into the graph space. The output features of the final layer of the segmentation network encoder are used as inputs to the FIGR module. For the input feature *F_input_*, its dimension size is *C*×*D*×*H*×*W*, where *C* represents the number of channels, and *D, H* and *W*, represent the depth, height, and width of the feature map, respectively. First, *F_input_* goes through two branches, and for the first branch, a convolution operation of 1×1×1 is performed on *F_input_* to adjust the number of channels from C to *C*/4. Subsequently, group normalization is performed on *F_input_* to accelerate the model convergence, and then a reshape operation is performed on *F_input_* to obtain the feature representation *θ*, *θ* ϵ R*^C^*^/4×(^*^HDW^*^)^. A Transpose operation is performed on *θ*. The dimension of *θ* is adjusted to *C*/4×(*HDW*). For the second branch, a convolution operation of 1×1×1 is performed on *F_input_* to adjust the number of channels from C to *C*/2, followed by a group normalization and reshape operation on *F_input_* to obtain the feature representation *φ*, *φ* ϵ R *^C^*^/2×(^*^HDW^*^)^. Matrix multiply *φ* with *θ* after the transpose operation to obtain the projected feature *V_ƒ_* ϵ R*^sf×sf^, S_f_* =*C*/2, *N_f_* =*C*/4 in the new coordinate space.

Graph Reasoning: With the graph projection from the previous step, the fully connected graph is constructed in feature space along the feature channels, the features are projected onto the graph nodes, and to capture the contextual dependencies between the nodes, graph convolution operations are required to propagate information and feature aggregation on the constructed fully connected graph. In the FIGR module, node information exchange and propagation are achieved through two Conv1Ds. Firstly, the Conv1D operation is passed in the node dimension in order to achieve the dissemination and aggregation of information among nodes. Secondly, the Conv1D operation in the channel dimension performs a weighting operation on the channels inside the node to capture the interactions between the channels. Thus, graphical reasoning can be expressed in Eq.(**10**) [30].

**Table:** 

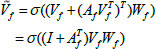	(10)

Where *V*^~^*_ƒ_* is the updated node feature representation, *V_ƒ_* is the feature representation of the input node, *A_ƒ_* is the adjacency matrix, *W_ƒ_* is the weight matrix, and *σ* is the activation function, *I* + *A^T^_ƒ_* is defined as an operation that enables Laplace smoothing [31, 32], which serves to smooth out node features as well as propagate information over the graph structure. Careful analysis shows the graph reasoning step's computational complexity hinges on the number of channels. Nodes exchange information to capture dependencies, with the adjacency matrix governing connections and the weight matrix updating states. As node interactions occur along the channel dimension, computation escalates with more channels.

Graph reprojection: After global relational reasoning by the FIGR module, the output feature graph is obtained. Since the output at the decoder side of the segmentation network is a regular convolutional feature, the output feature map after graph reasoning needs to be subjected to a reprojection operation to achieve a coordinate space consistent with the convolutional feature. The graph reprojection has four parts. First, the graph-reasoned feature map *V*^~^_ƒ_ϵR*^N^*^ƒ×^*^S^*^ƒ^ is fused with the previously graph-projected feature *θ*. This recovers and adjusts feature dimensionality and information representation, integrating features at different stages. Second, the new feature dimensions are adjusted to match the input feature maps' spatial dimensions for subsequent convolution operations. Third, new features are transformed and fused *via* convolution to restore the channel number to that of the input features, facilitating better fusion. Fourth, the convolved features are normalized for stable and consistent distribution, aiding feature fusion. Finally, the convolved and normalized features are residually joined with the original features. This preserves original information while enabling the network to capture richer representations, producing the final fused feature *F_out_*.

Moreover, to further showcase the efficacy of integrating the FIGR module before the attention module, let the input feature tensor be *X* ϵ R*^C^*^×^*^D^*^×^*^H^*^×^*^W^*, with C for channels, D for depth, H for height, and W for width. Denote the attention and FIGR modules as functions *A*(·) and *FIGR*(·). We use mutual information from information theory to quantify the information gain when they interact. *I*(*X*; *A*(*X*)) is the mutual info between *X* and *A*(*X*). *Y*= *FIGR*(*X*), then *I*(*Y*; *A*(*Y*)) is the mutual info after fusing FIGR before the attention module. Since FIGR enriches and reconstructs features *I*(*Y*; *A*(*Y*)) > *I*(*X*; *A*(*X*)). This shows that fusing FIGR helps the model get more valid input info, improving performance.

### 2.6 Total Loss

The proposed total loss of the network is calculated as shown in Eq. (**11**).

**Table:** 

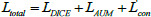	(11)

Where *L_total_* is the total loss in supervised and unsupervised training at multiple scales, *L_DICE_* is the loss of consistency between the multiscale output prediction and the ground truth of the student model in supervised training, *L_AUM_* is the cross-entropy loss between the probability distribution after resampling the multivariate normal distribution and the ground truth on a dual scale, and *L^’^_con_* is the final loss of the consistency loss guided by KL-variance as uncertainty estimation at multiple scales.

## 3 RESULTS

### 3.1 Dataset

This experiment uses the publicly available dataset MosMedData [12] provided by the municipal hospital in Moscow, Russia, which contains both labeled and unlabeled data, and the composition of the dataset used for training in the experiment is shown in Table **1**.

### 3.2 Evaluating Metrics

In order to assess the segmentation effect more effectively, four commonly used evaluation metrics were used in this experiment to quantitatively analyze the segmentation effect on lung lesions, including the Dice coefficient, Jaccard coefficient, normalized surface distance (NSD), and average distance of boundaries (ADB). Generally, the higher the score values of Dice, Jaccard, and NSD, the more accurate the segmentation of the lesion is, whereas for ADB, the lower the score value, the better the segmentation is.

Dice and Jaccard coefficients are calculated as shown in Eqs. (**12**) and (**13**) [23], both of which are generally used to evaluate the similarity between two sets by calculating the ratio between the number of common elements and the total number of elements in the two sets, and their values are usually between 0 and 1.

**Table:** 

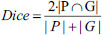	(12)

**Table:** 

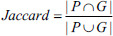	(13)

Where |*P*|, denotes the number of pixels in the prediction result and the number of pixels in the ground truth, respectively. |*P* Ո *G*| denotes the number of pixels that overlap between the predicted segmentation map and the ground truth, and |*P* U *G*| denotes the number of pixels in the concatenated portion of the two regions.

The NSD coefficient is usually used to evaluate the difference between the model's predicted results and the ground truth. The degree of similarity or overlap between the predicted results and the ground truth can be quantified by calculating the NSD coefficients, as shown in Eq. (**14**) [23].

**Table:** 

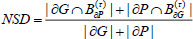	(14)

where *B_∂P_*^(^*^τ^*^)^ denotes the boundary portion or edge portion of the predicted surface and *B_∂G_*^(^*^τ^*^)^ denotes the boundary portion or edge portion of the ground truth.

The calculation of the ADB coefficient is based on the distance between the boundary of the prediction and the boundary of the ground truth, which can reflect the degree of boundary difference between the prediction result and the ground truth. The formula is shown in Eqs. (**15**-**17**) [23].

**Table:** 

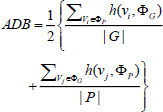	(15)

**Table:** 

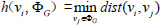	(16)

**Table:** 

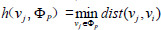	(17)

Where φ*_P_* denotes the surface of the segmentation result and φ*_G_* denotes the surface of the ground truth. *v_i_* and *v_j_* are the vertices denoting φ*_P_* and φ*_G_*, respectively. In addition, |*P*| and |*G*| have the same meaning as in Eqs. (**12**) and (**13**).

### 3.3 Experimental Setting

The Adam optimizer is used to adaptively optimize the network during training, and the initial value of the learning rate is set to 0.0003. The batch size is set to 1, and the total number of training rounds is 400 epochs. The decay factor for calculating the exponential moving average is set to 0.999, and the initial weight for consistency is set to a default value of 0.1. Data enhancement was performed in the experiments by adding noise and random flipping to the training set. The images were resized to 160×160×32 before feeding into the network, in addition, the random seed was set to 1337 to ensure the reproducibility of the experiment.

For this experiment, the network model was implemented with the utilization of the PyTorch deep-learning framework. The hardware setup included a GeForce RTX 3090 graphics card with 24GB of video memory, running on the Windows 10 operating system. The training time for our network model was 16 hours.

### 3.4 Ablation Studies

In order to verify the validity of the modules proposed by comparing the Dice coefficient, Jaccard coefficient, NSD, and ADB under different segmentation frameworks and network structures, to verify the effectiveness of different modules on the segmentation results, the results are shown in Table **2**. The results show that the two-stage dual multiscale uncertainty estimation and graph reasoning segmentation framework achieves 63.97%, 48.16%, 90.03%, and 3.27% on the four metrics coefficients, which is an improvement of 1.25%, 1.03%, 2.98%, and 0.59% over the baseline model, respectively. After using the aleatoric uncertainty module and uncertainty estimation alone on the baseline basis, both NSD and ADB metrics are improved, and after combining the two uncertainty estimations, compared to the baseline network, there is a better improvement in all four metrics. After integrating the graph reasoning module into the network, the network is more effective in segmenting the infected regions of the lungs, and the performance of the model reaches its optimal level.

### 3.5 Comparative Experiments

#### 3.5.1 Comparison Experiment of Adding Gaussian Noise at Different Locations

In order to improve the robustness and generalization ability of the model, this study adds an appropriate amount of Gaussian noise to the image data inputted into the teacher model and the student model, respectively, and the results are shown in Table **3**. The results show that the performance of the model is poorer when no noise is added to the image data input for both the teacher model and the student model. The effectiveness of the model was improved when noise was added only to the data entered into the teacher model. The model performs best when noise is added only to the data entered into the student model. When noise is added to the data entered into both the student model and the teacher model, the performance of the model decreases. Thus, the experiments demonstrated that the predictive distribution of the teacher model without noise input can be better fitted when only an appropriate amount of Gaussian noise is added to the image data input to the student model.

#### 3.5.2 Comparative Experiments with the Graph Reasoning Module at Different Locations

To further verify the effect of the FIGR module on the overall performance of the model at different positions, the FIGR module is added at the positions of the second, third, and fourth layers of the network, respectively, to verify the effect of different positions on the segmentation accuracy. The results are shown in Table **4**, where the number on the left represents the position of the layer where the FIGR module is located. When the FIGR module is added to the fourth layer, all four evaluation metrics are better than the effect in other positions, and the segmentation accuracy and the overall performance of the model at this time reach the optimum.

In addition, we compared the results achieved by the baseline model fused with the FIGR module with those of the popular Vision Transformer (VIT) [33] and Swin Transformer (W-MSA) [34] on the baseline model, and the results are shown in Fig. (**8a**). It is clear that the network incorporating the FIGR module achieves the best results and the model takes up the least number of parameters, as shown in Fig. (**8b**).

#### 3.5.3 Comparative Experiments for Modeling Uncertainty at Different Scales

Choosing to model aleatoric uncertainty at different scales with multivariate normal distributions may affect the overall segmentation effect. Therefore, to better optimize the performance of the semi-supervised segmentation algorithm, this study investigates the effect of modeling arbitrary uncertainty at different scales on the overall segmentation accuracy through comparative experiments. Fig. (**9a-d**) represent the modeling of arbitrary uncertainties at 1 to 4 scales. The results show that when aleatoric uncertainty is modeled simultaneously at the first and second scales, as shown in Fig. (**9b**), the segmentation accuracy of the lung lesions outperforms that of the other cases and leads to the best overall results.

#### 3.5.4 Comparative Experiments on the Number of Graph Nodes

In the task of lung infection region segmentation, a network incorporating a FIGR module has a relationship between the segmentation effect and the number of graph nodes. In order to verify the influence brought by the number of graph nodes on the overall segmentation performance during graph reasoning, the overall segmentation effect of the model is compared when the number of nodes is C/2, C/4, and C/8. The results are shown in Table **5**. It is clear that when the number of nodes is C/4, the values on all segmentation metrics are better than the values in the cases of C/2 and C/8 nodes. Therefore, too many or too few nodes will have an effect on the segmentation effect, and an appropriate number of nodes will enable the model to better capture the detailed features and global contextual information in the image.

#### 3.5.5 Comparison of Thresholding Methods with Uncertainty Estimation

In this study, we compared our multi-scale uncertainty estimation method for correcting noisy pseudo-label learning with the traditional approach of manually setting thresholds to filter low-quality pseudo-labels. As shown by multiple evaluation metrics in Table **6**, compared to the traditional method, our proposed method demonstrates superior performance in metrics such as DICE, Jaccard, NSD, and ADB, thus validating its effectiveness and superiority in correcting noisy pseudo-label learning.

#### 3.5.6 Comparative Experiments at Different Scales

Multiscale consistency constraints are used in both the supervised and unsupervised parts, and this study further validates the effect of multiscale consistency constraints on the overall segmentation effect. For this reason, this study constructs the consistency constraint framework based on one-scale, two-scale, three-scale, and four-scale, respectively, and the segmentation results are shown in Fig. (**10**). The results show that the supervised and unsupervised parts can achieve the best overall results while having a higher segmentation effect when using consistency constraints at all four scales simultaneously. This suggests that the consistency constraints at the dual multiscale help the segmentation method proposed to better identify the infected regions in pneumonia and to better mine useful information from unlabeled data.

### 3.6 Visualization of Segmentation Results

To vividly exhibit the segmentation effect, a set of segmentation results was randomly sampled from the test set for visualization. The 2D visualization results are presented in Fig. (**11**). The segmentation method proposed in this paper showcases remarkable accuracy in segmenting pneumonia lesions. It closely aligns with the ground truth, minimizing classification errors. Even when confronted with the MosMedData dataset, which contains small lung infection regions, the proposed method can accurately identify these small target areas. Notably, it can precisely segment the fuzzy-infected regions and their boundaries. As is manifest from the visualized 2D images, the proposed segmentation method outperforms in accurately identifying and outlining lesions. It accomplishes accurate segmentation for pneumonia lesions of diverse sizes. The overall shape and intricate details of large lesions are comprehensively depicted, while small lesions are precisely captured without any omissions or misclassifications.

## 4 DISCUSSION

### 4.1 Experimental Comparison

To validate the segmentation performance, the proposed method is compared with the state of the art segmentation networks as well as recent semi-supervised based segmentation methods, including DM^2^T-Net [23], UA-MT [17], Inf-Net [14], nn-UNet [35], 3D U-Net [36], TA-SagNet [37], *etc*. Table **7** shows the quantitative results for Dice, Jaccard, NSD, ADB, and HD95. As can be seen from the table, our proposed segmentation method has higher values than the 2D or 3D-based deep learning methods in the five evaluation metrics, which is because our proposed method can make full use of the unlabeled data during the training process and combine it with the labeled data to co-train the network model. And compared to other semi-supervised segmentation methods, the proposed method has higher values for Dice, Jaccard, and NSD, while achieving lower scores on the ADB metric. When conducting a comparison of the values in terms of the HD95 (95% Hausdorff Distance) metric between our newly proposed segmentation method and several widely recognized mainstream segmentation networks, it's crucial to note that in the context of the HD95 metric, lower values indicate superior segmentation performance. The tabular data vividly and incontrovertibly demonstrates that our method significantly surpasses the others in terms of the HD95 metric. Our approach yields notably lower HD95 values, thus clearly highlighting its enhanced segmentation capabilities. In this study, we added a vision transformer to the baseline MDA-CNN to build a vision transformer and multiple-dimensional attention convolutional neural network (VTMDA-CNN). Results on the MosMedData dataset show VTMDA-CNN underperforms. Likely, the limited data samples here, far fewer than ViT's ideal training amount, caused underfitting, hampering segmentation. In addition, the improvement over the baseline model DM^2^T-Net was 1.25%, 1.03%, 2.98%, and 0.59% on Dice, Jaccard, NSD, and ADB, respectively. This further suggests that the two-stage semi-supervised segmentation based on dual multiscale uncertainty estimation with graph reasoning in this paper produces more reliable predictions. Therefore, with limited labeling data, complex and variable lung lesion sizes, and ambiguous infected areas and boundaries, our method can still provide accurate identification of infected areas.

### 4.2 Discusses the Performance of GRMA-Net

We replaced the MosMedData dataset used for model training with another COVID-19-P20 [41] dataset that was not used for training and segmented the lung infection region in the COVID-19-P20 dataset to verify the generalization performance of the model, and the results are shown in Table **8**. The results show that our method has better values on Dice and higher scores on Jaccard and NSD, which indicates that the network performs better on the two cross-datasets with good generalization ability. In addition, the results of the network's segmentation of lung infections in the COVID-19-P20 dataset are shown in Fig. (**12**), where the area enclosed by the red line is the ground truth and the area enclosed by the green line is the prediction result.

In addition, we show some examples of incorrect segmentation and highlight the detailed information with boxes, as shown in Fig. (**13**). To distinguish the type of mis-segmentation, red and blue lines are used to indicate two different types of prediction results, while the green line represents the ground truth. Two scenarios lead to incorrect segmentation. First, in some cases where the characteristics of the normal lung tissue are very similar to those of the infected region, it is possible that the network may not be able to correctly discriminate between the infected region and the normal lung tissue region, which ultimately leads to incorrect segmentation, as shown by the region surrounded by the blue line in Fig. (**13**). Second, for the incorrect segmentation results shown in the other boxes, although there is also incorrect segmentation caused by the previous case, it is more often since the characteristics of the infected region are similar to the characteristics of the normal lung tissue region, which ultimately leads to the fact that it is not correctly classified as a diseased region. Therefore, to minimize erroneous segmentation, innovative methods like adaptive feature extraction [42], advanced attention mechanisms [43], and hierarchical k-fold cross-validation [44] offer valuable insights and guide future semi-supervised medical image segmentation research. Building on these, we aim to enhance the performance and applicability of semi-supervised methods in medical imaging, overcome existing challenges, and explore new frontiers.

## CONCLUSION

In order to fully explore and utilize the information in unlabeled data and reduce the impact of fuzzy infected regions or boundaries and noise-containing pseudo-labels on segmentation performance, we provide an in-depth analysis of the concepts of uncertainty estimation and graph convolution based on semi-supervised learning. A semi-supervised segmentation framework for uncertainty estimation and modeling at a two-stage, multi-scale is designed. Based on this framework, a FIGR module that can perform graph reasoning in the feature space along the feature channel is fused in front of the attention module of the network. In the process of supervised training, modeling aleatoric uncertainty as a multivariate normal distribution at two different scales, which can effectively capture the correlation between pixels and reduce the impact of blurred lesions and their boundaries on segmentation performance. In addition, guiding consistency loss with noisy pseudo-labels by calculating the uncertainty of teacher-student models at multiple scales, allowing the model to be fitted towards the correct target and reducing the problem of cognitive bias in the model while making full use of the unlabeled data for training. Finally, graph reasoning is incorporated into the network to effectively capture long-range dependencies between distant pixels, allowing the network to recognize small target regions with more finesse. The experimental results indicate that our proposed method achieved a Dice score of 63.97% on the MosMedData dataset. It shows that the proposed method has significant advantages, has important practical clinical significance, and can provide more effective assistance to assist physicians in making diagnoses. In future work, we will employ advanced generative adversarial network techniques to generate MosMedData-like synthetic image data. Although re-designing the network is challenging, we will strive for its successful implementation to simulate diverse datasets and comprehensively assess the robustness of our study results.

## AUTHORS’ CONTRIBUTIONS

The authors confirm their contribution to the paper as follows: J.W., Y.Z., S.T., Q.H.: Visualization; B.Z., J.L., D.Y.: Methodology; X.L. Validation; Y.G., J.Z.: Writing - Original Draft Preparation; L.Y., X.L.: Writing - Reviewing and Editing. All authors reviewed the results and approved the final version of the manuscript.

## Figures and Tables

**Fig. (1):**
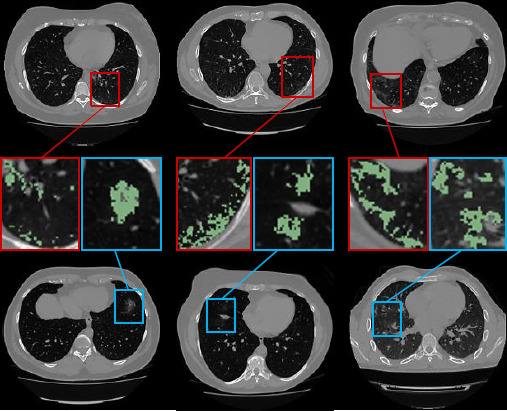
CT images of the lungs of a patient diagnosed with pneumonia. Available under the terms of the Creative Commons Attribution Non-Commercial License 4.0 [12].

**Fig. (2):**
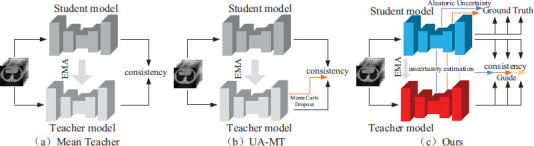
Classical mean teacher model (**a**), uncertainty-aware average teacher model (**b**), and the semi-supervised framework proposed in this paper (**c**).

**Fig. (3):**
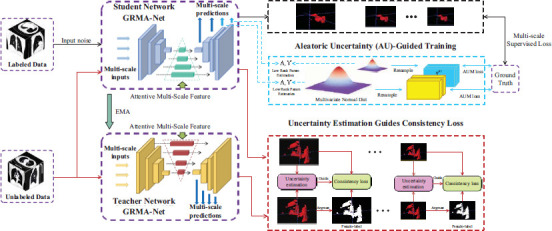
Dual multiscale uncertainty estimation and graph reasoning framework.

**Fig. (4):**
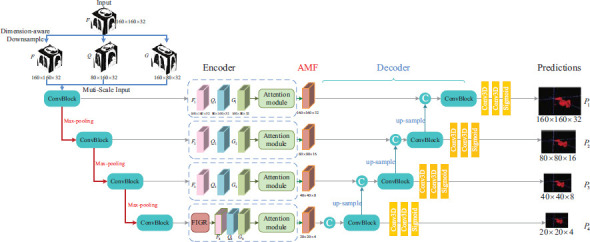
GRMA-Net structure.

**Fig. (5):**
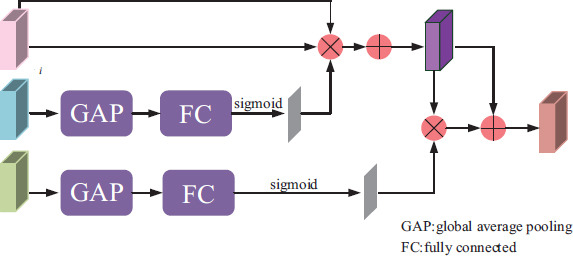
Attention module [23].

**Fig. (6):**
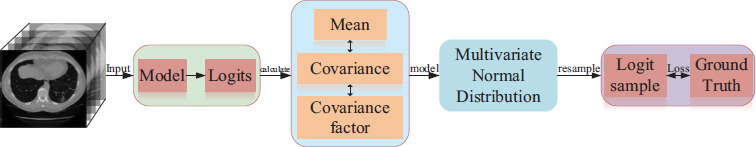
Aleatoric uncertainty modeling process.

**Fig. (7):**
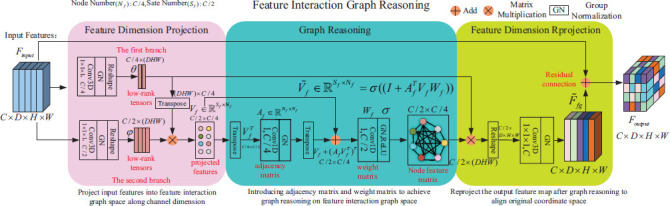
Details of the FIGR module [30].

**Fig. (8a, b):**
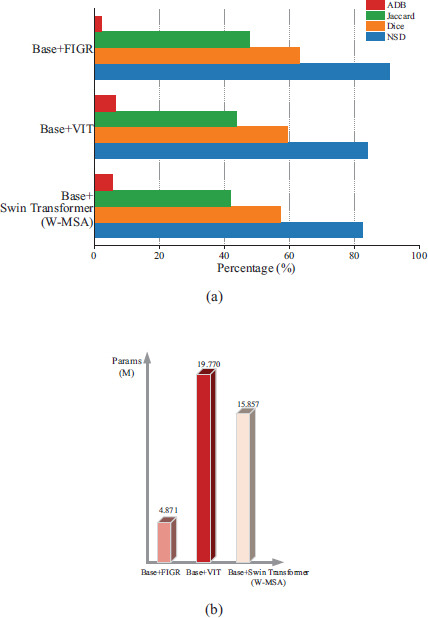
Comparison of effects and parameters with VIT and Swin Transformer (W-MSA).

**Fig. (9a-d):**
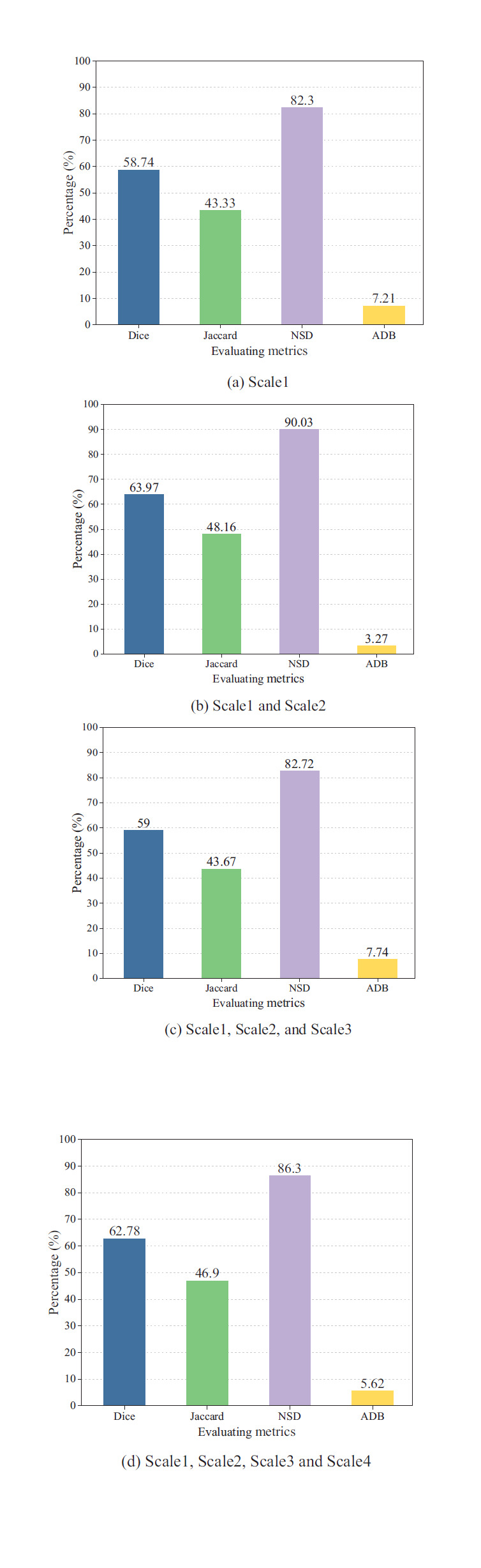
Comparative experiments with different numbers of AU modules.

**Fig. (10):**
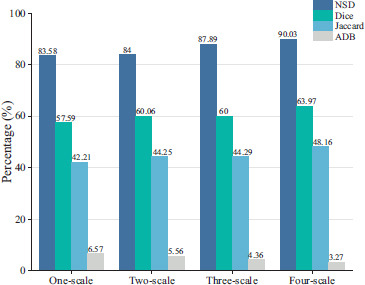
Comparative experiments at different scales.

**Fig. (11):**
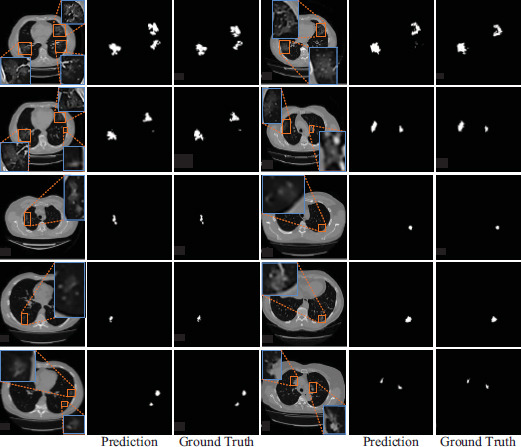
Visualization of 2D segmentation results.

**Fig. (12):**
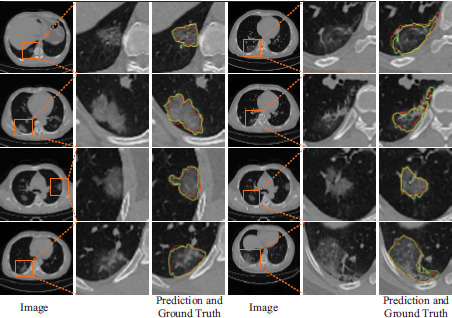
Segmentation results on the COVID-19-P20 dataset.

**Fig. (13):**
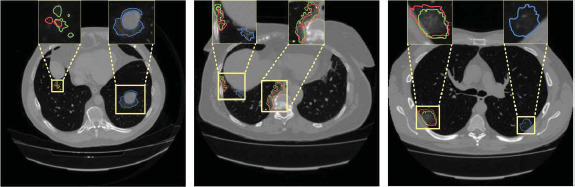
Example of erroneous segmentation.

**Table 1:** Composition of the dataset.

MosMedData	Train	Validation	Test
Labeled	32 cases	8 cases	10cases
Unlabeled	806 cases		

**Table 2:** Ablation study with semi-supervised segmentation algorithm (mean ± standard deviation).

**Method**	**BASE**	**Aleatoric Uncertainty**	**Uncertainty Estimation**	**Graph Reasoning**	**DICE**	**Jaccard**	**NSD**	**ADB**
BASE	√	**×**	**×**	**×**	62.71**±**13.21	47.13**±**15.12	87.05**±**11.51	3.86**±**4.35
BASE+AU	√	√	**×**	**×**	61.58**±**12.72	45.80**±**14.27	87.20**±**8.74	**2.93±3.51**
BASE+UE	√	**×**	√	**×**	62.30**±**11.80	46.41**±**13.57	87.56**±**8.09	3.86**±**3.87
BASE+GR	√	**×**	**×**	√	63.29**±**12.82	47.65**±**14.53	**90.87±6.38**	2.33**±**2.44
BASE+AU+UE	√	√	√	**×**	63.96**±**10.67	47.97**±**12.21	88.91**±**6.12	3.65**±**4.22
BASE+AU+UE+GR	√	√	√	√	**63.97±11.36**	**48.16±13.59**	90.03**±**5.59	3.27**±**3.96

**Table 3:** Comparative experiments for different locations of noise.

**Evaluating metrics (Mean ± Standard Deviation)**				
Student	Teacher	DICE	Jaccard	NSD
×	×	60.44**±**13.75	44.77**±**14.87	86.01**±**11.05
×	√	63.00**±**11.50	47.08**±**13.18	90.70**±**5.62
√	×	**63.97±11.36**	**48.16±13.59**	**90.03±5.59**
√	√	62.51±12.95	46.80±14.17	85.76±13.65

**Table 4:** Comparative experiments of the graph reasoning module at different locations.

**Evaluating Metrics (Mean ± Standard Deviation)**				
Layer	DICE	Jaccard	NSD	ADB
2	59.87**±**15.06	44.42**±**15.97	83.94**±**14.10	6.35**±**8.10
3	58.93**±**15.24	43.41**±**15.26	82.00**±**16.58	7.50**±**8.09
4	**63.97±11.36**	**48.16±13.59**	**90.03±5.59**	**3.27±3.96**

**Table 5:** Comparative experiments on the number of graph nodes.

**Evaluating Metrics (Mean ± Standard Deviation)**				
Node Number	DICE	Jaccard	NSD	ADB
C/2	62.87±10.91	46.86±12.78	87.83±6.67	4.11±3.77
C/4	**63.97±11.36**	**48.16±13.59**	**90.03±5.59**	**3.27±3.96**
C/8	62.60±12.48	46.85±14.22	89.47±5.80	3.57±3.59

**Table 6:** Comparison of thresholding methods with uncertainty estimation.

**Evaluating Metrics (mean)**					
**Methods**	**Threshold**	**DICE**	**Jaccard**	**NSD**	**ADB**
Pseudo-labeling	>0.60	60.46	44.81	85.29	5.75
Pseudo-labeling	>0.70	62.31	46.27	88.81	3.02
Pseudo-labeling	>0.80	61.88	46.08	87.02	4.80
Ours	-	**63.97**	**48.16**	**90.03**	3.27

**Table 7:** Comparison of results with other models and algorithms (**\** denote not mentioned).

**Method/Refs.**	**Data Type**	**DICE**	**Jaccard**	**NSD**	**ADB**	**HD95**
GAS net [38]	3D	54.20±22.4	\	\	\	\
GAN + UNet + Contrastive [39]	2D+3D	58.43±19.0	\	\	\	\
Inf-Net [14]	2D	51.8±0.31	\	\	\	\
TA-SegNet [37]	2D	62.30±0.18	\	\	\	\
U-Net++ [40]	2D	56.03±22.46	42.05±20.39	77.71±22.71	5.51±6.94	22.05±28.17
3D U-Net [36]	3D	54.42±23.51	40.69±20.70	75.31±27.09	8.21±14.43	21.35±27.39
nn-UNet [35]	3D	56.30±23.55	42.62±21.31	76.45±27.16	9.26±18.25	20.59±29.97
UA-MT [17]	3D	57.31±20.53	42.87±19.05	78.55±21.42	6.89±13.90	20.68±26.53
DM^2^T-Net [23]	3D	60.19±19.22	45.56±18.44	80.95±20.99	6.55±13.79	21.11±27.74
VTMDA-CNN	3D	58.85±16.23	43.49±15.92	81.47±15.98	7.70±8.06	31.96±30.46
Our method	3D	**63.97±11.36**	**48.16±13.59**	**90.03±5.59**	**3.27±3.96**	**16.84±25.67**

**Table 8:** Generalization analysis.

**MosMedData COVID-19-P20**			
**Method/Refs.**	**Dice**	**Jaccard**	**NSD**
U-Net++ [40]	23.61±17.31	-	-
UA-MT [17]	50.24±17.70	-	-
DM^2^T-Net [23]	51.36±17.80	-	-
Ours	**62.43±20.50**	**48.56±21.43**	**86.12±12.75**

## Data Availability

The data that support the findings of this study are openly available in the MosMedData dataset at https://doi.org/ 10.48550/arXiv.2005.06465.

## LIST OF ABBREVIATIONS

AU: Aleatoric Uncertainty
FIGR: Feature Interaction Graph Reasoning
NSD: Normalized Surface Dice
ADB: Average Distance of Boundaries
CT: Computed Tomography
CAD: Computer-Aided Diagnosis
RCTE: Reliable and Consistent Temporal-Ensembling
MT: Mean Teacher
KL: Kullback-Leibler
EMA: Exponential Moving Average
UA: Uncertainty-Aware
CNN: Convolutional Neural Network
GRMA-Net: Graph Reasoning and Multi-dimensional Attention Network
MDA-CNN: Multiple-Dimensional Attention Convolutional Neural Network
VTMDA-CNN: Vision Transformer and Multiple-Dimensional Attention Convolutional Neural Network
GAP: Global Average Pooling
FC: Fully Connected
VIT: Vision Transformer
W-MSA: Window Multi-head Self-Attention
HD95: 95% Hausdorff Distance

## References

[1] Haider N.S., Behera A.K. (2024). Computerized respiratory sound based diagnosis of pneumonia.. Med. Biol. Eng. Comput..

[2] Torres A., Cilloniz C., Niederman M.S., Menéndez R., Chalmers J.D., Wunderink R.G., van der Poll T. (2021). Pneumonia.. Nat. Rev. Dis. Primers.

[3] Rajaraman S., Candemir S., Kim I., Thoma G., Antani S. (2018). Visualization and interpretation of convolutional neural network predictions in detecting pneumonia in pediatric chest radiographs.. Appl. Sci..

[4] Aydoğdu M., Ozyilmaz E., Aksoy H., Gürsel G., Ekim N. (2010). Mortality prediction in community-acquired pneumonia requiring mechanical ventilation; values of pneumonia and intensive care unit severity scores.. Tuberk. Toraks.

[5] Dong D., Fang M.J., Tang L., Shan X.H., Gao J.B., Giganti F., Wang R.P., Chen X., Wang X.X., Palumbo D., Fu J., Li W.C., Li J., Zhong L.Z., De Cobelli F., Ji J.F., Liu Z.Y., Tian J. (2020). Deep learning radiomic nomogram can predict the number of lymph node metastasis in locally advanced gastric cancer: An international multicenter study.. Ann. Oncol..

[6] Dong D., Zhang F., Zhong L.Z., Fang M.J., Huang C.L., Yao J.J., Sun Y., Tian J., Ma J., Tang L.L. (2019). Development and validation of a novel MR imaging predictor of response to induction chemotherapy in locoregionally advanced nasopharyngeal cancer: A randomized controlled trial substudy (NCT01245959).. BMC Med..

[7] Gu Y., Lu X., Zhang B., Zhao Y., Yu D., Gao L., Cui G., Wu L., Zhou T. (2019). Automatic lung nodule detection using multi-scale dot nodule-enhancement filter and weighted support vector machines in chest computed tomography.. PLoS One.

[8] Lakhani P., Sundaram B. (2017). Deep learning at chest radiography: Automated classification of pulmonary tuberculosis by using convolutional neural networks.. Radiology.

[9] Lu X., Gu Y., Yang L., Zhang B., Zhao Y., Yu D., Zhao J., Gao L., Zhou T., Liu Y., Zhang W. (2020). Multi-level 3D densenets for false-positive reduction in lung nodule detection based on chest computed tomography.. Curr. Med. Imaging.

[10] Wang B., Li M., Ma H., Han F., Wang Y., Zhao S., Liu Z., Yu T., Tian J., Dong D., Peng Y. (2019). Computed tomography-based predictive nomogram for differentiating primary progressive pulmonary tuberculosis from community-acquired pneumonia in children.. BMC Med. Imaging.

[11] Gu Y., Chi J., Liu J., Yang L., Zhang B., Yu D., Zhao Y., Lu X. (2021). A survey of computer-aided diagnosis of lung nodules from CT scans using deep learning.. Comput. Biol. Med..

[12] Morozov S.P., Andreychenko A.E., Pavlov N.A. (2020). Mosmeddata: Chest ct scans with covid-19 related findings dataset.. medRXiv.

[13] Feng X., Lin J., Feng C.M., Lu G. (2024). GAN inversion-based semi-supervised learning for medical image segmentation.. Biomed. Signal Process. Control.

[14] Fan D.P., Zhou T., Ji G.P., Zhou Y., Chen G., Fu H., Shen J., Shao L. (2020). Inf-net: Automatic covid-19 lung infection segmentation from ct images.. IEEE Trans. Med. Imaging.

[15] Jiao R., Zhang Y., Ding L., Xue B., Zhang J., Cai R., Jin C. (2024). Learning with limited annotations: A survey on deep semi-supervised learning for medical image segmentation.. Comput. Biol. Med..

[16] Tarvainen A., Valpola H. (2017). Mean teachers are better role models: Weight-averaged consistency targets improve semi-supervised deep learning results.. Adv. Neural Inf. Process. Syst..

[17] Yu L, Wang S, Li X, Fu CW, Heng PA (2019). Uncertainty-aware self-ensembling model for semi-supervised 3D left atrium segmentation.. Medical Image Computing and Computer Assisted Intervention – MICCAI 2019.

[18] Lu L., Yin M., Fu L., Yang F. (2023). Uncertainty-aware pseudo-label and consistency for semi-supervised medical image segmentation.. Biomed. Signal Process. Control.

[19] Xiang J., Qiu P., Yang Y., Wang L, Dou Q, Fletcher PT, Speidel S, Li S (2022). FUSSNet: Fusing two sources of uncertainty for semi-supervised medical image segmentation.. Medical Image Computing and Computer Assisted Intervention – MICCAI 2022.

[20] Lei T., Zhang D., Du X., Wang X., Wan Y., Nandi A.K. (2023). Semi-supervised medical image segmentation using adversarial consistency learning and dynamic convolution network.. IEEE Trans. Med. Imaging.

[21] Wu H., Wang Z., Song Y., Yang L., Qin J. Cross-patch dense contrastive learning for semi-supervised segmentation of cellular nuclei in histopathologic images.. Proceedings of the IEEE/CVF Conference on Computer Vision and Pattern Recognition.

[22] Wang K., Zhan B., Zu C., Wu X., Zhou J., Zhou L., Wang Y. (2022). Semi-supervised medical image segmentation *via* a tripled-uncertainty guided mean teacher model with contrastive learning.. Med. Image Anal..

[23] Wang L., Wang J., Zhu L., Fu H., Li P., Cheng G., Feng Z., Li S., Heng P.A. (2023). Dual multiscale mean teacher network for semi-supervised infection segmentation in chest CT volume for COVID-19.. IEEE Trans. Cybern..

[24] Gu Y., Lu X., Yang L., Zhang B., Yu D., Zhao Y., Gao L., Wu L., Zhou T. (2018). Automatic lung nodule detection using a 3D deep convolutional neural network combined with a multi-scale prediction strategy in chest CTs.. Comput. Biol. Med..

[25] Monteiro M., Le Folgoc L., Coelho de Castro D., Pawlowski N., Marques B., Kamnitsas K., van der Wilk M., Glocker B. (2020). Stochastic segmentation networks: Modelling spatially correlated aleatoric uncertainty.. Adv. Neural Inf. Process. Syst..

[26] Wang Y., Xiao B., Bi X., Li W., Gao X. MCF: Mutual correction framework for semi-supervised medical image segmentation.. Proceedings of the IEEE/CVF Conference on Computer Vision and Pattern Recognition.

[27] Sohn K., Berthelot D., Carlini N., Zhang Z., Zhang H., Raffel C.A., Cubuk E.D., Kurakin A., Li C.L. (2020). Fixmatch: Simplifying semi-supervised learning with consistency and confidence.. Adv. Neural Inf. Process. Syst..

[28] Luo X, Liao W, Chen J, Song T, Chen Y, Zhang S, Chen N, Wang G, Zhang S (2021). Efficient semi-supervised gross target volume of nasopharyngeal carcinoma segmentation *via* uncertainty rectified pyramid consistency.. Medical Image Computing and Computer Assisted Intervention – MICCAI 2021.

[29] Zheng Z., Yang Y. (2021). Rectifying pseudo label learning *via* uncertainty estimation for domain adaptive semantic segmentation.. Int. J. Comput. Vis..

[30] Zhuang Y., Liu H., Song E., Hung C.C. (2023). A 3D cross-modality feature interaction network with volumetric feature alignment for brain tumor and tissue segmentation.. IEEE J. Biomed. Health Inform..

[31] Chen Y., Rohrbach M., Yan Z., Shuicheng Y., Feng J., Kalantidis Y. Graph-based global reasoning networks.. 2019 IEEE/CVF Conference on Computer Vision and Pattern Recognition (CVPR).

[32] Liu Z., Tong L., Chen L., Zhou F., Jiang Z., Zhang Q., Wang Y., Shan C., Li L., Zhou H. (2021). Canet: Context aware network for brain glioma segmentation.. IEEE Trans. Med. Imaging.

[33] Dosovitskiy A., Beyer L., Kolesnikov A. (2020). An image is worth 16x16 words: Transformers for image recognition at scale.. arXiv:2010.11929.

[34] Liu Z., Lin Y., Cao Y., Hu H., Wei Y., Zhang Z., Lin S., Guo B. (2021). Swin transformer: Hierarchical vision transformer using shifted windows.. arXiv:2103.14030.

[35] Isensee F., Petersen J., Kohl S.A., Jäger P.F., Maier-Hein K.H. (2019). nnu-net: Breaking the spell on successful medical image segmentation.. https://www.researchgate.net/publication/332494163_nnU-Net_Breaking_the_Spell_on_Successful_Medical_Image_Segmentation.

[36] Çiçek Ö, Abdulkadir A, Lienkamp SS, Brox T, Ronneberger O, Ourselin S, Joskowicz L, Sabuncu M, Unal G (2016). 3D U-Net: Learning dense volumetric segmentation from sparse annotation.. Medical Image Computing and Computer-Assisted Intervention – MICCAI 2016.

[37] Mahmud T., Alam M.J., Chowdhury S., Ali S.N., Rahman M.M., Anowarul Fattah S., Saquib M. (2021). CovTANet: A hybrid tri-level attention-based network for lesion segmentation, diagnosis, and severity prediction of COVID-19 chest CT scans.. IEEE Trans. Industr. Inform..

[38] Xu Z., Cao Y., Jin C., Shao G., Liu X., Zhou J., Shi H., Feng J.J. (2020). Gasnet: Weakly-supervised framework for covid-19 lesion segmentation.. https://www.researchgate.net/publication/344757137_GASNet_Weakly-supervised_Framework_for_COVID-19_Lesion_Segmentation.

[39] Shabani S., Homayounfar M., Vardhanabhuti V., Nikouei Mahani M.A., Koohi-Moghadam M. (2022). Self-supervised region-aware segmentation of COVID-19 CT images using 3D GAN and contrastive learning.. Comput. Biol. Med..

[40] Zhou Z., Rahman Siddiquee M.M., Tajbakhsh N., Liang J. UNet++: A nested U-Net architecture for medical image segmentation.. Proceedings of the 4th International Workshop.

[41] Ma J., Wang Y., An X., Ge C., Yu Z., Chen J., Zhu Q., Dong G., He J., He Z., Cao T., Zhu Y., Nie Z., Yang X. (2021). Toward data‐efficient learning: A benchmark for COVID‐19 CT lung and infection segmentation.. Med. Phys..

[42] Alhussen A., Anul Haq M., Ahmad Khan A., Mahendran R.K., Kadry S. (2025). XAI-RACapsNet: Relevance aware capsule network-based breast cancer detection using mammography images *via* explainability O-net ROI segmentation.. Expert Syst. Appl..

[43] Khan A.A., Madendran R.K., Thirunavukkarasu U., Faheem M. (2023). *D*
^2^
*PAM* : Epileptic seizures prediction using adversarial deep dual patch attention mechanism.. CAAI Trans. Intell. Technol..

[44] Kujur A, Raza Z, Khan AA (2022). Data complexity based evaluation of the model dependence of brain MRI images for classification of brain tumor and Alzheimer’s disease.. IEEE Access.

